# Editorial: Advances in breeding for wheat disease resistance

**DOI:** 10.3389/fpls.2022.1034506

**Published:** 2022-09-16

**Authors:** Christina Cowger, Peter Bulli, Feng Chen, Morten Lillemo, Marco Maccaferri

**Affiliations:** ^1^ United States Department of Agriculture, Plant Science Research Unit, Raleigh, NC, United States; ^2^ School of Agricultural and Food Sciences, Jaramogi Oginga Odinga University of Science and Technology, Bondo, Kenya; ^3^ National Key Laboratory of Wheat and Corn Crop Science/Agronomy College, Centro de Investigation y Mejoramiento de Maiz y Trigo-China Wheat and Maize Joint Research Center, Henan Agricultural University, Zhengzhou, China; ^4^ Department of Plant Sciences, Norwegian University of Life Sciences, Ås, Norway; ^5^ Department of Agricultural and Food Sciences, University of Bologna, Bologna, Italy

**Keywords:** wheat, disease resistance, breeding, QTL mapping, magic, cereal rusts, Fusarium head blight, powdery mildew

Wheat is the most widely planted crop on the planet and contributes up to 20% of total calorie intake for humankind. Maintaining wheat yields is crucial to feeding the world’s people, especially as climate models suggest that rising global temperatures will negatively affect wheat production (Asseng et al., 2015). Diseases of wheat take an important toll, annually robbing humanity of 20% or more of the crop on a global basis (Savary et al., 2019; Savary and Willocquet, 2021). Changes in weather patterns may accelerate pathogen life cycles and escalate shifts in pathogen populations and virulence, posing significant challenges to disease resistance breeding. As well, global trade may increase the chances for a pathogen to spread rapidly and adapt to novel environments and even hosts, leading to emerging diseases.

The release and use of wheat cultivars with effective and durable disease resistance is more important now than ever. This is so for multiple reasons. First, disease resistance stabilizes yields and reduces economic losses, saving money for producers who are already facing major challenges due to rising temperatures, more frequent and unpredictable natural disasters, and high and rising costs of inputs such as pesticides (FAO, 2021; Lüttringhaus et al., 2021; Miedaner and Juroszek, 2021). Second, greater reliance on disease resistance can slow pathogen spread and multiplication, prolonging the useful life of available pesticide chemistries so they will be effective when needed to manage severe epidemics (Brent et al., 2007). Third, the growing use of conservation tillage, which is vital for soil health and stabilization, has elevated the importance of diseases such as Fusarium head blight that cannot be completely managed with fungicides (Aboukhaddour et al., 2020).

Breeding for disease resistance in wheat has made major technological advances, but still faces important challenges. Prominent among those challenges is the need to develop cultivars for a tremendous diversity of agro-ecological environments, production practices, and discrete market classes (Cowger, 2021). Another challenge is that major genes such as those traditionally deployed to manage wheat rust diseases are often rapidly overcome. This requires a focus on quantitative and race non-specific resistance that may be harder to introgress, select for, and retain in a multi-trait context (Cowger and Brown, 2019; van Esse et al., 2020). The more genes are identified and their mechanisms of action elucidated, the more tools will be available to researchers and breeders to assemble genetically novel germplasm with improved and more durable resistance.

The authors who have contributed to this Research Topic tackle those challenges by providing new resources and tools to aid wheat breeders across the globe. The 18 original articles cover a good sample of the world’s most important wheat diseases and the state-of-the-art techniques applied by researchers to identify and evaluate the relevant disease resistance traits. For example, wheat blast is an emergent and damaging disease that has jumped continents from Latin America to Asia, as explained in a comprehensive review by Singh et al., 2021. A team of blast researchers has compared marker-assisted and genomic selection using precision phenotyping of blast resistance conferred by the 2NS translocation (Juliana et al., 2022), which is partial and sometimes background-dependent.

Another major threat to global wheat production is Fusarium head blight. Three articles in this Research Topic offer important new resources for breeding cultivars with effective FHB resistance. The Brazilian spring cultivar Surpresa provides a new source of resistance not currently used (Poudel et al. 2022). Three resistance loci (*Fhb1*, *Fhb4*, and *Fhb5*) were introgressed as a pyramid into desirable Chinese white and red semi-winter wheat lines (Zhang et al., 2021). And a novel technique could speed up the development of FHB-resistant winter wheat germplasm, increasing breeding generations from two to three per year (Zakieh et al. 2021).

Researchers used various approaches to identify new sources of resistance to the three wheat rusts (stem, stripe, and leaf). A new stem rust resistance gene was mapped in the durum wheat variety Kronos and introgressed into common wheat using co-segregating DNA markers (Li et al., 2021). The effects of combinations of leaf rust resistance genes were investigated in a Canadian wheat double-haploid population (McCallum and Hiebert, 2022) and in a durably resistant Canadian wheat cultivar (Bokore et al., 2022). A multi-parent advanced generation intercross (MAGIC) wheat population was used to map adult-plant and seedling resistance to stripe rust in Germany (Rollar et al., 2021). A genome-wide association study was used to identify stripe rust resistance loci in a panel of Chinese wheat landraces (Yao et al., 2021). And QTL mapping led to identification of stripe and leaf rust loci in an Afghan landrace (Zhang et al., 2022), a Chinese landrace (Wang et al., 2022), and the CIMMYT wheat line “Mucuy” (Lan et al., 2022; so far this is an abstract, need the URL to the full article when it’s available).

Breeding wheat cultivars with resistance to powdery mildew requires a constant stream of new resistance sources, thanks to the pathogen’s ability to rapidly overcome host resistance through adaptation. The efficacy of a set of new resistance genes introgressed from Middle Eastern wild wheat relatives was measured using powdery mildew populations from various wheat growing regions affected by the disease (Kloppe et al., 2022). A more unusual wild relative of wheat, *Psathyrostachys huashanica*, which is found only in the Huashan Mountains of China, also furnished novel resistance to wheat powdery mildew (Liu et al., 2021).

A previously unidentified source of resistance to Hessian fly was identified in spring wheat cultivars of the U.S. Pacific Northwest (Prather et al., 2022). And in a twist, a locus conferring not resistance but susceptibility, in this case to tan spot, was identified in U.S. bi-parental spring wheat mapping populations and narrowed to a region encompassing seven candidate genes (Running et al., 2022). Last but not least, an interesting look under the ground revealed that rhizosphere microbiomes differed among wheat genotypes and had an impact on pathogenicity of *Rhizoctonia solani*, suggesting the potential to manage Rhizoctonia root rot with wheat genotypes that recruit microbiomes associated with improved plant fitness and suppression of the fungal pathogen (Dilla-Ermita et al., 2021).

For this Research Topic, we have collected articles that demonstrate how cutting-edge approaches to breeding are being brought to bear on some of the chief diseases threatening the world’s wheat production systems. The authors’ contributions are of the highest quality, and illustrate the strong international interest in this topic. These reports help breeders everywhere assess and employ novel and potentially durable resistance to wheat diseases. They will make a practical difference in helping safeguard global wheat yields in the challenging years to come.

## Author contributions

CC drafted the editorial and all authors helped edit it. All authors contributed to the article and approved the submitted version.

## Funding

Research in the laboratories of the Topic Editors is supported by the U.S. Department of Agriculture – Agricultural Research Service (CC); the National Research Fund Kenya (PB); the National Key Research and Development Program of China (2019YFE0118300) (FC); the Research Council of Norway and the Foundation for Research Levy on Agricultural Products (FFL) and the Agricultural Agreement Research Fund (JA) in Norway (ML); and the EU H2020 and PRIMA European projects (MM).

## Conflict of interest

The authors declare that the research was conducted in the absence of any commercial or financial relationships that could be construed as a potential conflict of interest.

## Publisher’s note

All claims expressed in this article are solely those of the authors and do not necessarily represent those of their affiliated organizations, or those of the publisher, the editors and the reviewers. Any product that may be evaluated in this article, or claim that may be made by its manufacturer, is not guaranteed or endorsed by the publisher.
